# Association of Menopause and Hormonal Contraceptive Use With Chronic Rhinosinusitis: An “All of Us” Analysis

**DOI:** 10.1002/ohn.70067

**Published:** 2025-11-12

**Authors:** Abraham Ahn, Richard G. Chiu, Kamal Eldeirawi, Anthony I. Dick, Sharmilee M. Nyenhuis, Victoria S. Lee

**Affiliations:** ^1^ Department of Otolaryngology College of Medicine, University of Illinois Chicago Chicago Illinois USA; ^2^ Department of Population Health Nursing Science College of Nursing, University of Illinois Chicago Chicago Illinois USA; ^3^ Department of Pediatrics, Section of Allergy and Immunology University of Chicago Chicago Illinois USA

**Keywords:** biomarkers, chronic rhinosinusitis, CRS, female, hormonal contraceptives, menopause

## Abstract

**Objective:**

The role of sex hormones in inflammation has attracted increased attention, but the impact of hormone‐modifying states in chronic rhinosinusitis (CRS) has not been thoroughly studied. We aim to investigate the association of menopause and systemic hormonal contraceptive (SHC) use with both CRS with (CRSwNP) and without (CRSsNP) nasal polyps.

**Study Design:**

Cross‐sectional analysis of data.

**Setting:**

A large, national dataset of adults from the *All of Us* Research Program.

**Methods:**

This study was conducted using data from 239,564 participants. CRS diagnoses, biomarker data, menopause status, and SHC use, including both estrogen‐containing contraceptives (ECCs) and progestin‐only contraceptives (POCs), were extracted. Multivariable logistic and linear regression models were constructed to analyze the associations of CRS diagnosis and biomarkers with menopause and SHC use.

**Results:**

Postmenopausal participants had no significant difference in CRS odds compared to premenopausal patients (odds ratio [OR]: 1.02; 95% confidence interval [CI]: 0.94‐1.11). Compared to no SHC use, POCs were not associated with CRS, but ECC use was associated with a lower odds of CRSsNP (OR: 0.71; 95% CI: 0.64‐0.80) while being not associated with CRSwNP (OR: 0.82; 95% CI: 0.51‐1.25). Biomarker analysis revealed that ECC use was associated with a decreased serum neutrophil concentration among participants without CRS (*β*: −1.29; 95% CI: −1.72 to −0.85) and among participants with CRSsNP (*β*: −1.66; 95% CI: −3.02 to −0.29) compared to no SHC use.

**Conclusion:**

Menopause was not associated with CRS, while ECC use was associated with a lower odds of CRSsNP. Further research is needed to clarify underlying mechanisms.

Chronic rhinosinusitis (CRS) is a condition characterized by persistent inflammation of the nasal and paranasal sinus mucosa lasting at least 12 weeks. It presents with key symptoms such as nasal obstruction, drainage, impaired sense of smell, and facial pain or pressure. This condition significantly impacts quality of life, affecting approximately 2.1% to 13.8% of adults in the United States (US) and accounting for over $10 billion in annual healthcare costs.^1^ CRS is classified into 2 phenotypes based on the presence (CRSwNP) or absence (CRSsNP) of nasal polyps. Its pathogenesis involves various inflammatory pathways, with CRSsNP primarily driven by a neutrophilic T‐helper 1 (Th1) response. On the contrary, CRSwNP is associated with an eosinophilic T‐helper 2 (Th2) response. These pathways underlie endotypic classifications of CRS determined based on disease biomarkers. The type 2 endotype is more strongly linked to CRSwNP and includes biomarkers such as eosinophils, immunoglobulin E (IgE), interleukin (IL)‐4, IL‐5, IL‐13, matrix metallopeptidases (MMPs), and leukotriene E4 (LTE4). Non‐type 2 endotypes are more heterogeneous in their phenotypic presentation and includes biomarkers such as neutrophils, IL‐17A, interferon (IFN)‐γ, tumor necrosis factor (TNF)‐α, and uric acid.^2^, ^3^

The role of sex hormones such as estrogen and progesterone has been attracting increased attention in conditions such as CRS based on their effects on inflammatory pathways. Specifically, progesterone and testosterone have demonstrated anti‐inflammatory effects systemically, while estrogen has shown varied proinflammatory and anti‐inflammatory effects.^4^, ^5^, ^6^ Moreover, the presence of estrogen receptors in the nasal mucosa could provide a possible mechanism for anticipating an effect of sex hormones on sinonasal disease.^7^, ^8^, ^9^, ^10^ One study investigating the expression of these estrogen receptors among women taking oral contraceptives even demonstrated a decrease of beta‐receptors in lamina propria cells.^11^ Studies of primary sinonasal malignancies have also found prominent expression of these sex hormone receptors.^12^, ^13^ For these reasons, we sought to better understand the association of hormone‐modifying states such as menopause and systemic hormonal contraceptive (SHC) use with a diagnosis of CRS. We define SHCs as any form of contraception that acts through systemic mechanisms, thereby affecting the entire body, as opposed to those which act locally within the reproductive tract. Menopause marks the transition when women experience a significant decline in estrogen and progesterone production. Similarly, SHCs, which can be classified into progestin‐only contraceptives (POCs) and estrogen‐containing contraceptives (ECCs), may result in alterations in sex hormone levels through the direct addition of exogenous synthetic hormones, negative feedback mechanisms, and production of binding proteins.

Primary literature investigating the effect of menopause and SHC use on CRS among adult females is sparse. A systematic review examining literature in 2 databases ranging from 1966 to 2012 found that there were no studies relating menopause or contraceptives with rhinosinusitis.^14^ One study discovered that postmenopausal women had prolonged nasal mucociliary clearance time but did not investigate how menopause affects CRS odds.^15^ However, multiple studies have established an association between CRS and impairment in mucociliary clearance owing to possible mechanisms such as reduction in ciliary beat frequency and alteration of airway secretions.^16^, ^17^ Another study investigated the association of hormone replacement therapy (HRT) with CRS among postmenopausal women; however, this study assessed the association of HRT with the utilization of antibiotics and endoscopic sinus surgery (ESS) rather than disease odds.^18^ Moreover, postmenopausal patients on HRT have a much different hormonal state than premenopausal patients taking SHCs.

Given the differential effects of estrogen and progesterone on systemic inflammation, we sought to investigate the association of SHCs and menopause with a diagnosis of CRS and associated biomarkers. To our knowledge, no other study has explicitly examined the connection between menopause, SHC use, and CRS odds among US adults, and this is the first to do so using data from the All of Us Research Program (AoURP).

## Methods

### Study Population

This study used data from AoURP's Controlled Tier Dataset v8, available to authorized users on the Researcher Workbench. AoURP is a national US dataset aimed at targeting adult populations that are underrepresented in biomedical research, collecting data through surveys, electronic health records (EHR), biosamples, and physical measurements.^19^ Participants are enrolled through partner healthcare organizations, such as academic medical centers, Veterans Affairs facilities, and community health centers. All participants are aged 18 or older at the time of enrollment. Given that AoURP is a de‐identified public dataset, the study was deemed exempt by the University of Illinois Chicago institutional review board.

AoURP v8 includes data for 395,987 biological females in the United States, of whom 239,564 had EHR data available. These participants with EHR data were initially considered for inclusion; however, participants with missing data for any variable considered in our analysis were excluded. We conducted 2 separate analyses for this study, one investigating the association of menopause and CRS, and the other investigating SHC use and CRS. Each analysis had distinct inclusion and exclusion criteria to best isolate the exposure and remove potential confounders. For the menopause analysis, we only included females aged 40 to 60 years to best isolate the perimenopausal period and excluded individuals who were taking hormonal medications. For the SHC analysis, we only considered females aged 20 to 40 to best isolate the reproductive period and excluded individuals who had reached menopause. For both analyses, patients who were pregnant at the time of data collection or had a history of oophorectomy were excluded. Flow charts for cohort selection are included in the Supplementary Materials (Supplemental Figures S1 and S2, available online).

### Outcome Variables, Exposure Variables, and Covariates

The primary outcome of interest in our study was participants' CRS diagnosis, which was divided into CRSsNP and CRSwNP. Medical diagnoses in AoURP are based on medical records and are identified using Systemized Nomenclature of Medicine (SNOMED) and International Classification of Diseases (ICD)9 and ICD10 codes. AoURP standardizes these source codes into a unified vocabulary and classification system. To identify CRS diagnoses, we selected conditions classified under “chronic sinusitis” or “polyp of nasal cavity and/or nasal sinus.” These conditions encompass ICD‐9 code categories 473 and 471, respectively, and ICD‐10 code categories J32 and J33, respectively. A complete list of ICD and SNOMED codes are included in the Supplementary Materials (Supplemental Table S1, available online). CRS diagnoses were categorized as CRSwNP if the participant's diagnosis included the term “polyp,” with remaining diagnoses classified as CRSsNP. To support these associations, laboratory data on CRS biomarkers including serum levels of eosinophils, neutrophils, IgE, IFN‐γ, TNF‐α, IL‐4, IL‐5, IL‐13, IL‐17A, uric acid, and MMP9, as well as nasal nitric oxide and urine LTE4, were additionally aggregated and analyzed as an additional outcome.

Our study investigated 2 primary variables of interest, namely participant menopause status and SHC use. Menopause status was determined using participant responses to the survey questions “Have your menstrual periods stopped permanently?” and “Why did your periods stop?”. SHC use was determined using the “Hormonal contraceptives for systemic use” variable within the AoURP Workbench, which aggregates any lifetime exposures to SHCs recorded in participants' electronic health records. For patients with CRS, we only included SHCs which were initiated prior to the date of CRS diagnosis to best isolate temporally relevant exposures. A histogram distribution of time between SHC initiation and date of CRS diagnosis is included in our Supplementary Materials (Supplemental Figure S3, available online). These SHCs were then subclassified into POC or ECC based on drug name. We only considered contraceptives with high systemic absorption in our analysis, specifically pills, patches, vaginal rings, and injections. Participants using intrauterine devices, inserts, creams, and sprays were placed in the group with no SHC use, based on evidence indicating lower systemic hormone absorption with these contraceptive methods.^20^, ^21^, ^22^, ^23^ A sensitivity analysis completely removing participants taking contraceptives with low systemic absorption was also conducted to inform the validity of our findings. Participants only taking emergency contraceptive pills without other SHC use were additionally considered to have no SHC use given the single‐use nature of emergency contraception.

While SHCs should only include progestin‐only or combination estrogen and progestin drugs, some estrogen‐only medications were classified as SHCs in AoURP. This may be due to data misclassification or mislabeling of combined hormonal contraceptives. We chose to include these estrogen‐only drugs in the analysis alongside ECCs and performed an additional sensitivity analysis excluding these drugs completely.

Covariates included in our analysis were demographics (age, race/ethnicity), socioeconomic status (income, education, and insurance status), and relevant risk factors and comorbidities (smoking history, asthma, gastroesophageal reflux disease [GERD]) based on prior literature supporting the association between these factors and CRS.^24^, ^25^

### Statistical Analysis

Participant characteristics were summarized for both menopause and SHC cohorts, with chi‐square and t‐tests used to compare groups by menopausal status and SHC use, respectively. Multivariable logistic regression models were then used to evaluate the independent association of menopause and SHC use with all CRS, and stratified into CRSsNP and CRSwNP, with separate models being constructed for each analysis. Adjusted odds ratios (OR) and 95% confidence intervals (CI) were calculated.

We additionally performed an exploratory analysis assessing the association of menopause and SHC use with CRS biomarkers to support our primary outcomes. We first determined the sample size for each biomarker in both the menopause and SHC cohorts. Biomarkers with sample size over 20 were included as an outcome variable in a multiple linear regression model to examine the adjusted association between either menopause or SHC use and CRS biomarkers. A sample size of 20 was selected based on literature suggesting that only 2 subjects per predictor variable are needed to estimate regression coefficients with low relative bias in linear regression models.^26^ Separate models were constructed for each biomarker and for each cohort stratified by CRS status. For each model, *β* and 95% CI were calculated.

A *P* < .05 was used to denote statistical significance. Analyses were conducted using R version 4.4.0 (R Foundation for Statistical Computing) within the AoURP Workbench cloud analysis environment.

## Results

### Menopause Analysis

Participant characteristics and bivariate analyses for the menopause analysis cohort are included in Table 1. A total of 38,749 participants met the inclusion and exclusion criteria for this analysis, of which 4348 participants (11.2%) had CRS. The mean age of this study cohort was 50.5 years, and participants were predominantly non‐Hispanic White (52.8%), earned more than 50,000 USD (51.4%), received college education (53.0%), and were insured through private/employer plans (56.6%). After stratification by menopause status, 17,043 participants (44.0%) had not undergone menopause and 21,706 (56.0%) had. In terms of biomarkers, postmenopausal patients had higher levels of serum eosinophils and uric acid, while having a lower concentration of serum neutrophils (*P* < .001). Postmenopausal participants were more likely to have a diagnosis of CRS, including both CRSsNP and CRSwNP, be older age, non‐Hispanic White, have an income less than 25,000 USD, be insured by private/employer plans, and have a diagnosis of asthma or GERD compared to premenopausal women (all *P* < .001). Postmenopausal women were also less likely to have obtained a graduate/professional degree compared to premenopausal women (*P* < .001).

**Table 1:** Participant Characteristics of Cohort for Menopause Analysis After Inclusion/Exclusion Criteria

		Menopause status		
Variable	Full cohort (N = 38,749)	Premenopausal (N = 17,043)	Postmenopausal (N = 21,706)	*P*‐value
No CRS, n (%)	34,401 (88.8%)	15,314 (89.9%)	19,087 (87.9%)	<.001*
CRS, n (%)	4348 (11.2%)	1729 (10.1%)	2619 (12.1%)	
CRSsNP	4100 (10.6%)	1627 (9.5%)	2473 (11.4%)	<.001*
CRSwNP	248 (0.6%)	102 (0.6%)	146 (0.7%)	<.001*
Serum biomarkers, mean (SD)				
Eosinophils (% of WBCs)	2.14 (2.03)	2.03 (1.97)	2.23 (2.06)	<.001*
Neutrophils (% of WBCs)	62.29 (11.77)	63.24 (11.43)	61.57 (11.97)	<.001*
IgE (IU/mL)	167.51 (433.37)	164.37 (352.87)	170.10 (490.19)	.835
Uric acid (mg/dL)	4.92 (1.72)	4.76 (1.56)	5.03 (1.81)	<.001*
Age, mean (SD)	50.5 (5.9)	46.6 (4.5)	53.6 (4.8)	<.001*
Race/ethnicity, n (%)				<.001*
Non‐Hispanic White	20,470 (52.8%)	8360 (49.1%)	12,110 (55.8%)	
Non‐Hispanic Black	7822 (20.2%)	3401 (20.0%)	4421 (20.4%)	
Hispanic	7125 (18.4%)	3631 (21.3%)	3494 (16.1%)	
Other	3332 (8.6%)	1651 (9.7%)	1681 (7.7%)	
Annual income (USD), n (%)				<.001*
Less than 10,000	6778 (17.5%)	2897 (17.0%)	3881 (17.9%)	
10,000‐25,000	5639 (14.6%)	2283 (13.4%)	3356 (15.5%)	
25,000‐35,000	3081 (8.0%)	1401 (8.2%)	1680 (7.7%)	
35,000‐50,000	3326 (8.6%)	1513 (8.9%)	1813 (8.4%)	
50,000‐75,000	4612 (11.9%)	1994 (11.7%)	2618 (12.1%)	
75,000‐150,000	9005 (23.2%)	3943 (23.1%)	5062 (23.3%)	
More than 150,000	6308 (16.3%)	3012 (17.7%)	3296 (15.2%)	
Education, n (%)				<.001*
Less than high school	3316 (8.6%)	1456 (8.5%)	1860 (8.6%)	
High school or GED	6599 (17.0%)	2704 (15.9%)	3895 (17.9%)	
Any college	20,536 (53.0%)	8783 (51.5%)	11,753 (54.1%)	
Graduate/professional	8298 (21.4%)	4100 (24.1%)	4198 (19.3%)	
Health Insurance, n (%)				<.001*
None	2771 (7.2%)	1399 (8.2%)	1372 (6.3%)	
Medicare/Medicaid	12,087 (31.2%)	4919 (28.9%)	7163 (33.0%)	
Private/Employer	21,917 (56.6%)	9923 (58.2%)	11,999 (55.3%)	
Other	1974 (5.1%)	802 (4.7%)	1172 (5.4%)	
Smoking History, n (%)				<.001*
No	23,612 (60.9%)	10,897 (63.9%)	12,715 (58.6%)	
Yes	15,137 (39.1%)	6146 (36.1%)	8991 (41.4%)	
Asthma, n (%)				<.001*
No	31,348 (80.9%)	13,975 (82.0%)	17,373 (80.0%)	
Yes	7401 (19.1%)	3068 (18.0%)	4333 (20.0%)	
GERD, n (%)				<.001*
No	28,184 (72.7%)	13,071 (76.7%)	15,113 (69.6%)	
Yes	10,565 (27.3%)	3972 (23.3%)	6593 (30.4%)	

*P*‐values calculated using chi‐square or *t*‐test as appropriate comparing postmenopausal participants with premenopausal participants.

Abbreviations: GED, general educational development; SD, standard deviation; USD, US Dollars.

* *P* < .05.

Results of multivariable logistic regression analysis for the association between menopause and CRS are shown in Table 2. After controlling for covariates, menopause was not associated with CRS (OR: 1.02; 95% CI: 0.94‐1.11), which remained true for both CRSsNP (OR: 1.03; 95% CI: 0.94‐1.12) and CRSwNP (OR: 0.90; 95% CI: 0.65‐1.23). In terms of covariates, CRS was independently associated with a middle‐range income of 35,000 to 50,000 USD, a high school or college‐level education, and Medicare/Medicaid or private employer insurance plans. Participants of non‐White race/ethnicity had a lower odds of CRS compared to non‐Hispanic White participants. Participants with asthma (OR: 2.76; 95% CI: 2.57‐2.97) and GERD (OR: 2.99; 95% CI: 2.79‐3.20) had a much greater odds of CRS compared to those without these diagnoses.

**Table 2:** Adjusted Associations Between Menopause and CRS

	CRS	CRSsNP	CRSwNP
Menopause			
No	Ref	Ref	Ref
Yes	1.02 (0.94‐1.11)	1.03 (0.94‐1.12)	0.90 (0.65‐1.23)
Age	1.01 (0.99‐1.01)	1.00 (0.99‐1.01)	1.02 (0.99‐1.05)
Race/ethnicity			
Non‐Hispanic White	Ref	Ref	Ref
Non‐Hispanic Black	**0.74 (0.67‐0.81)**	**0.72 (0.66‐0.80)**	0.96 (0.66‐1.38)
Hispanic	**0.62 (0.56‐0.69)**	**0.60 (0.53‐0.66)**	1.11 (0.77‐1.57)
Other	**0.70 (0.61‐0.79)**	**0.69 (0.60‐0.79)**	0.79 (0.46‐1.29)
Annual income (USD)			
Less than 10,000	Ref	Ref	Ref
10,000‐25,000	1.06 (0.94‐1.21)	1.05 (0.92‐1.19)	1.33 (0.78‐2.28)
25,000‐35,000	1.15 (0.99‐1.34)	1.10 (0.94‐1.29)	**2.13 (1.19‐3.83)**
35,000‐50,000	**1.28 (1.09‐1.49)**	**1.24 (1.05‐1.45)**	**2.08 (1.12‐3.87)**
50,000‐75,000	1.16 (0.99‐1.36)	1.13 (0.96‐1.33)	1.77 (0.94‐3.33)
75,000‐150,000	1.07 (0.92‐1.25)	1.04 (0.89‐1.22)	1.55 (0.84‐2.91)
More than 150,000	0.91 (0.77‐1.08)	0.88 (0.74‐1.04)	1.61 (0.82‐3.19)
Education			
Less than high school	Ref	Ref	Ref
High school or GED	**1.18 (1.01‐1.39)**	**1.19 (1.01‐1.40)**	0.96 (0.50‐1.94)
Any college	**1.31 (1.13‐1.53)**	**1.30 (1.11‐1.52)**	1.54 (0.86‐2.99)
Graduate/professional	1.16 (0.98‐1.38)	1.16 (0.97‐1.38)	1.21 (0.62‐2.51)
Health insurance			
None	Ref	Ref	Ref
Medicare/Medicaid	**1.25 (1.05‐1.48)**	**1.24 (1.05‐1.48)**	1.47 (0.72‐3.55)
Private/employer	**1.50 (1.25‐1.80)**	**1.49 (1.24‐1.79)**	1.95 (0.93‐4.80)
Other	1.14 (0.91‐1.43)	1.13 (0.90‐1.42)	1.48 (0.58‐4.05)
Smoking History			
No	Ref	Ref	Ref
Yes	0.93 (0.87‐1.00)	0.93 (0.87‐1.00)	0.94 (0.71‐1.23)
Asthma			
No	Ref	Ref	Ref
Yes	**2.76 (2.57‐2.97)**	**2.63 (2.44‐2.83)**	**6.03 (4.61‐7.92)**
GERD			
No	Ref	Ref	Ref
Yes	**2.99 (2.79‐3.20)**	**2.99 (2.79‐3.21)**	**2.76 (2.11‐3.62)**

Values represent OR (95% CI).

Abbreviations: CI, confidence interval; GED, general educational development; OR, odds ratio; USD, US Dollars.

Bold values denote *P* < .05.

Analysis of CRS biomarkers for the menopause analysis is shown in Table 3. Serum eosinophils, neutrophils, IgE, and uric acid had a sufficient sample size >20 for inclusion in regression analysis. A complete list of biomarkers and their sample sizes is included in our Supplementary Materials (Supplemental Table S2, available online). Menopause was associated with a higher concentration of serum eosinophils (*β*: 0.12; 95% CI: 0.06‐0.18) and uric acid (*β*: 0.14; 95% CI: 0.01‐0.27), but lower concentration of serum neutrophils (*β*: −1.24; 95% CI: −1.60 to −0.88) among the entire cohort. After stratification by CRS status, menopause was associated with an increased concentration of serum eosinophils among participants without CRS (*β*: 0.11; 95% CI: 0.04‐0.17) and those with CRSsNP (*β*: 0.18; 95% CI: 0.01‐0.36). On the contrary, menopause was associated with a lower concentration of serum neutrophils among participants without CRS (*β*: −1.19; 95% CI: −1.58 to −0.79) and those with CRSsNP (*β*: −1.33; 95% CI: −2.22 to −0.44). There was no association between menopause and either eosinophils (*β*: −0.13; 95% CI: −1.14‐0.88) or neutrophils (β: −0.95; 95% CI: −5.68‐3.78) among those with CRSwNP. There was also no statistically significant association between menopause and IgE or serum uric acid among those with or without CRS.

**Table 3:** Association of Menopause With CRS Biomarkers Stratified by CRS Status

Outcome	Full cohort	Strata of CRS status			
		No CRS	CRS	CRSsNP	CRSwNP
Serum eosinophils (% of WBCs)	N = 23,519	N = 19,966	N = 3553	N = 3360	N = 193
	**0.12 (0.06‐0.18)**	**0.11 (0.04‐0.17)**	0.16 (−0.01‐0.33)	**0.18 (0.01‐0.36)**	−0.13 (−1.14 to −0.88)
Serum neutrophils (% of WBCs)	N = 23,222	N = 19,691	N = 3531	N = 3337	N = 194
	**−1.24 (−1.60 to −0.88)**	**−1.19 (−1.58 to −0.79)**	**−1.33 (−2.21 to −0.46)**	**−1.33 (−2.22 to −0.44)**	−0.95 (−5.68‐3.78)
Serum IgE* (IU/mL)	N = 943	N = 616	N = 327	N = 285	N = 42
	−1.18 (−68.08 to 65.71)	23.58 (−66.93‐114.08)	−66.33 (−161.78‐29.13)	−78.39 (−180.97‐24.19)	−70.38 (−402.70‐261.94)
Serum uric acid* (mg/dL)	N = 3868	N = 2983	N = 885	N = 834	N = 51
	**0.14 (0.01‐0.27)**	0.12 (−0.02‐0.27)	0.19 (−0.07‐0.45)	0.20 (−0.08‐0.47)	0.81 (−0.45‐2.07)

N represents sample size, and bottom cell values represent *β* (95% CI) of postmenopausal women compared to premenopausal participants as the reference group, controlling for covariates.

Abbreviations: CI, confidence interval; CRS, chronic rhinosinusitis.

Bold values denote *P* < .05.

### SHC Analysis

Characteristics and bivariate analyses for the SHC analysis cohort are included in Table 4. We identified 44,280 participants who met the inclusion and exclusion criteria for this analysis, of which 3652 participants (8.2%) had CRS. The mean age was 31.0 years and participants were predominantly non‐Hispanic White (50.6%), college‐educated (57.3%), and insured through private/employer plans (59.7%). After stratification by SHC use, 35,076 participants (79.2%) did not use any SHCs, 3308 (7.5%) used POCs, and 5896 (13.3%) used ECCs. Participants with POC use were more likely to have a diagnosis of CRS (CRSsNP and CRSwNP) compared to those without SHC use (*P* = .012), but participants with ECC use had a lower prevalence of CRS (*P* = .019), explained primarily by CRSsNP. In terms of biomarkers, participants with POC use had a lower concentration of serum neutrophils compared to those without any SHC use (*P* = .014). Those with ECC use had a higher concentration of serum eosinophils (*P* = .022) but a lower concentration of neutrophils (*P* < .001), IgE (*P* = .006), and uric acid (*P* = .002). Participants with POC use were more likely to be older age (*P* = .002), non‐Hispanic Black, in lower income brackets, educated at a high school level, insured by Medicare/Medicaid, and have a history of smoking (*P* < .001). On the contrary, those with ECC use were more likely to be non‐Hispanic White, in higher income brackets, insured through private/employer plans, have a graduate/professional degree, and have no smoking history (*P* < .001). Participants with either POC and ECC use were more likely to have a diagnosis of asthma or GERD compared to those without SHC use (*P* < .001).

**Table 4:** Baseline Characteristics of Cohort for Systemic Hormonal Contraceptive Analysis After Inclusion/Exclusion Criteria

Variable	Full cohort (N = 44,280)	Hormonal contraceptives for systemic use^a^		
		None (N = 35,076)	POC (N = 3308)	ECC (N = 5896)
No CRS, n (%)	40,628 (91.8%)	32,174 (91.7%)	2992 (90.5%)	5462 (92.6%)
CRS, n (%)	3652 (8.2%)	2902 (8.3%)	316 (9.5%)	434 (7.4%)
CRSsNP	3480 (7.8%)	2774 (7.9%)	296 (8.9%)	410 (7.0%)
CRSwNP	172 (0.4%)	128 (0.4%)	20 (0.6%)	24 (0.4%)
*P*‐value	‐	‐	.012*	.019*
Serum biomarkers, mean (SD)				
Eosinophils (% of WBCs)	1.83 (1.95)	1.81 (1.96)	1.87 (1.87)	1.88 (1.90)
*P*‐value	‐	‐	.128	.022*
Neutrophils (% of WBCs)	63.81 (12.25)	64.15 (12.28)	63.49 (12.31)	62.52 (12.06)
*P*‐value	‐	‐	.014*	<.001*
IgE (IU/mL)	189.81 (535.76)	208.12 (609.90)	198.82 (318.31)	123.33 (278.28)
*P*‐value	‐	‐	.828	.006*
Uric acid (mg/dL)	4.65 (1.39)	4.68 (1.42)	4.66 (1.36)	4.51 (1.24)
*P*‐value	‐	‐	.774	.002*
Age, mean (SD)	31.0 (5.5)	31.0 (5.5)	31.3 (5.3)	30.8 (5.4)
*P*‐value	‐	‐	.002*	<.001*
Race/ethnicity, n (%)				
Non‐Hispanic White	22,415 (50.6%)	17,102 (48.8%)	1595 (48.2%)	3718 (63.1%)
Non‐Hispanic Black	6294 (14.2%)	4861 (13.9%)	933 (28.2%)	500 (8.5%)
Hispanic	10,258 (23.2%)	8734 (24.9%)	496 (15.1%)	1028 (17.4%)
Other	5313 (12.0%)	4379 (12.5%)	284 (8.6%)	650 (11.0%)
*P*‐value	‐	‐	<.001*	<.001*
Annual income (USD), n (%)				
Less than 10,000	7650 (17.3%)	6169 (17.6%)	803 (24.3%)	679 (11.5%)
10,000‐25,000	5727 (12.9%)	4558 (13.0%)	547 (16.5%)	622 (10.5%)
25,000‐35,000	4924 (11.1%)	3948 (11.3%)	386 (11.7%)	590 (10.0%)
35,000‐50,000	5629 (12.7%)	4532 (12.9%)	377 (11.4%)	720 (12.2%)
50,000‐75,000	6365 (14.4%)	5022 (14.3%)	386 (11.6%)	957 (16.3%)
75,000‐150,000	9334 (21.1%)	7284 (20.8%)	554 (16.7%)	1496 (25.4%)
More than 150,000	4651 (10.5%)	3563 (10.2%)	256 (7.7%)	832 (14.1%)
*P*‐value	‐	‐	<.001*	<.001*
Education, n (%)				
Less than high school	2228 (5.0%)	1841 (5.2%)	200 (6.0%)	187 (3.2%)
High school or GED	7310 (16.5%)	5900 (16.8%)	776 (23.5%)	634 (10.8%)
Any college	25,393 (57.3%)	20,095 (57.3%)	1801 (54.4%)	3497 (59.3%)
Graduate/professional	9349 (21.1%)	7240 (20.6%)	531 (16.1%)	1578 (26.8%)
*P*‐value	‐	‐	<.001*	<.001*
Health insurance, n (%)				
None	2580 (5.8%)	2139 (6.1%)	259 (7.8%)	182 (3.1%)
Medicare/Medicaid	12,714 (28.7%)	10,117 (28.9%)	1266 (38.3%)	1299 (22.0%)
Private/Employer	26,442 (59.7%)	20,650 (58.9%)	1658 (50.1%)	4154 (70.5%)
Other	2544 (5.7%)	2170 (6.2%)	125 (3.8%)	261 (4.4%)
*P*‐value	‐	‐	<.001*	<.001*
Smoking history, n (%)				
No	33,039 (74.6%)	26,098 (74.4%)	2286 (69.1%)	4655 (79.0%)
Yes	11,241 (25.4%)	8978 (25.6%)	1022 (30.9%)	1241 (21.0%)
*P*‐value	‐	‐	<.001*	<.001*
Asthma, n (%)				
No	36,841 (83.2%)	29,609 (84.4%)	2515 (76.0%)	4717 (80.0%)
Yes	7439 (16.8%)	5467 (15.6%)	793 (24.0%)	1179 (20.0%)
*P*‐value	‐	‐	<.001*	<.001*
GERD, n (%)				
No	36,610 (82.7%)	29,581 (84.3%)	2356 (71.2%)	4673 (79.3%)
Yes	7670 (17.3%)	5495 (15.7%)	952 (28.8%)	1223 (20.7%)
*P*‐value	‐	‐	<.001*	<.001*

Abbreviations: GED, general educational development; SD, standard deviation; USD, US Dollars.

* *P* < .05. *P*‐values calculated using chi‐square or *t*‐test as appropriate comparing each group to the cohort with no systemic hormonal contraceptive use.

^a^ Values only consider contraceptives initiated prior to CRS diagnosis in participants with CRS.

Multivariable logistic regression analyzing the association between SHC use and CRS are shown in Table 5. Participants with ECC use had a lower odds of having CRS (OR: 0.72; 95% CI: 0.65‐0.80), but POC use was not significantly associated with CRS (OR: 0.88; 95% CI: 0.77‐1.01). After stratification into CRSsNP and CRSwNP, ECC use was associated with a lower odds of CRSsNP (OR: 0.71; 95% CI: 0.64‐0.80) but not CRSwNP (OR: 0.82; 95% CI: 0.51‐1.25). For covariates, when compared to White participants, any non‐White race/ethnicity was associated with a lower odds of CRSsNP, while Hispanic and Other race/ethnicity were associated with a lower odds of CRSwNP. High school or college education and having insurance were associated with a higher odds of CRSsNP. Diagnoses of asthma and GERD were associated with a higher odds of both CRSsNP and CRSwNP. Our sensitivity analysis, included in our Supplementary Materials (Supplemental Tables S3 and S4, available online), revealed no statistically significant differences in our primary outcome when removing drugs listed as estrogen‐only in AoURP, as well as when we removed patients who were only taking non‐systemic contraceptives from the analysis entirely.

**Table 5:** Adjusted Associations Between Progestin‐Only or Estrogen‐Containing Systemic Hormonal Contraceptives and CRS, With Participants Without Systemic Hormonal Contraceptive Use as the Reference Group, CRS Stratified Into CRSsNP and CRSwNP

	All CRS	CRSsNP	CRSwNP
Hormonal contraceptive			
None	Ref	Ref	Ref
Progestin only	0.88 (0.77‐1.01)	0.87 (0.76‐1.00)	1.09 (0.65‐1.73)
Estrogen‐containing	**0.723 (0.65‐0.80)**	**0.71 (0.64‐0.80)**	0.82 (0.51‐1.25)
Age	**1.05 (1.04‐1.05)**	**1.05 (1.04‐1.06)**	1.01 (0.98‐1.04)
Race/Ethnicity			
Non‐Hispanic White	Ref	Ref	Ref
Non‐Hispanic Black	**0.68 (0.61‐0.77)**	**0.67 (0.59‐0.75)**	1.02 (0.65‐1.57)
Hispanic	**0.58 (0.53‐0.65)**	**0.59 (0.53‐0.65)**	**0.57 (0.36‐0.87)**
Other	**0.56 (0.49‐0.64)**	**0.57 (0.50‐0.65)**	**0.29 (0.12‐0.59)**
Annual Income			
Less than 10,000	Ref	Ref	Ref
10,000‐25,000	1.14 (0.99‐1.31)	1.12 (0.97‐1.29)	1.52 (0.86‐2.71)
25,000‐35,000	**1.17 (1.01‐1.36)**	1.15 (0.99‐1.34)	1.52 (0.82‐2.82)
35,000‐50,000	**1.16 (1.00‐1.35)**	1.16 (0.99‐1.35)	1.19 (0.60‐2.33)
50,000‐75,000	1.15 (0.99‐1.34)	1.14 (0.97‐1.33)	1.33 (0.68‐2.61)
75,000‐150,000	1.13 (0.97‐1.31)	1.11 (0.95‐1.29)	1.65 (0.87‐3.16)
More than 150,000	0.96 (0.80‐1.14)	0.93 (0.78‐1.12)	1.75 (0.82‐3.72)
Education			
Less than high school	Ref	Ref	Ref
High school or GED	**1.37 (1.10‐1.71)**	**1.38 (1.11‐1.73)**	1.21 (0.49‐3.62)
Any college	**1.60 (1.30‐1.98)**	**1.59 (1.29‐1.98)**	1.75 (0.75‐5.11)
Graduate/professional	1.22 (0.97‐1.55)	1.22 (0.97‐1.55)	1.26 (0.48‐3.94)
Health Insurance, n (%)			
None	Ref	Ref	Ref
Medicare/Medicaid	**1.33 (1.09‐1.63)**	**1.31 (1.07‐1.61)**	1.83 (0.75‐6.08)
Private/Employer	**1.50 (1.23‐1.86)**	**1.50 (1.22‐1.86)**	1.60 (0.63‐5.40)
Other	**1.37 (1.07‐1.75)**	**1.36 (1.06‐1.75)**	1.63 (0.51‐6.20)
Smoking History			
No	Ref	Ref	Ref
Yes	0.94 (0.86‐1.02)	0.94 (0.86‐1.03)	0.84 (0.57‐1.21)
Asthma			
No	Ref	Ref	Ref
Yes	**2.82 (2.61‐3.05)**	**2.71 (2.50‐2.93)**	**6.25 (4.54‐8.65)**
GERD			
No	Ref	Ref	Ref
Yes	**2.90 (2.69‐3.13)**	**2.90 (2.68‐3.14)**	**2.85 (2.06‐3.93)**

Values represent OR (95% CI).

Abbreviations: CI, confidence interval; GED, general educational development; OR, odds ratio; USD, US Dollars.

Bold values denote *P* < .05.

Regression analysis for the adjusted association between SHC use and CRS biomarkers is shown in Table 6. Among the full cohort, POC use was not associated with any biomarker, while ECC use was associated with a lower concentration of serum neutrophils (*β*: −1.27; 95% CI: −1.68 to −0.86) and uric acid (*β*: −0.17; 95% CI: −0.29 to −0.06). After stratification by CRS status, SHCs were not associated with serum eosinophils or IgE among participants with or without CRS. While POCs were not associated with serum neutrophils among those with or without CRS, ECCs were associated with a decreased concentration of serum neutrophils among patients without CRS (*β*: −1.29; 95% CI: −1.72 to −0.85) and those with CRSsNP (*β*: −1.66; 95% CI: −3.02 to −0.29), but not among those with CRSwNP. ECC use was also associated with lower serum uric acid levels among participants without CRS (*β*: −0.17; 95% CI: −0.30 to −0.05), while not being associated among those with CRS.

**Table 6:** Association of Systemic Hormonal Contraceptive Use With CRS Biomarkers Stratified by CRS Status

Outcome	Full cohort	Strata of CRS status			
		No CRS	CRS	CRSsNP	CRSwNP
Serum eosinophils (% of WBCs)	N = 25,549	N = 22,658	N = 2891	N = 2751	N = 140
	0.03 (−0.05‐0.12)	0.05 (−0.03‐0.14)	−0.10 (−0.37‐0.17)	−0.15 (−0.42‐0.12)	0.07 (−1.69‐1.82)
	0.04 (−0.02‐0.11)	0.05 (−0.02‐0.12)	0.02 (−0.21‐0.25)	0.065 (−0.18‐0.28)	−0.58 (−2.11‐0.95)
Serum Neutrophils (% of WBCs)	N = 25,277	N = 22,412	N = 2865	N = 2726	N = 139
	−0.21 (−0.74‐0.31)	−0.37 (−0.92‐0.19)	0.80 (−0.74‐2.33)	0.55 (−1.04‐2.13)	6.17 (−0.39‐12.73)
	**−1.28 (−1.69 to −0.87)**	**−1.29 (−1.72 to −0.85)**	**−1.61 (−2.93 to −0.29)**	**−1.66 (−3.02 to −0.29)**	−0.82 (−6.53‐4.89)
Serum IgE* (IU/mL)	N = 932	N = 658	N = 274	N = 245	N = 29
	−9.34 (−133.78‐115.09)	−48.86 (−217.61‐119.89)	76.50 (−73.53‐226.53)	20.41 (−144.78‐185.61)	71.95 (−839.43‐983.33)
	−66.13 (−152.23‐19.97)	−100.44 (−212.88‐ 11.99)	11.18 (−103.09‐125.45)	−25.79 (−152.19‐100.61)	183.92 (−38.11‐405.96)
Serum uric acid* (mg/dL)	N = 4,344	N = 3,716	N = 628	N = 597	N = 31
	−0.06 (−0.19‐0.08)	−0.02 (−0.17‐0.13)	**−0.42 (−0.79 to −0.04)**	−0.36 (−0.75‐0.02)	−0.96 (−3.24‐1.31)
	**−0.17 (−0.29 to −0.06)**	**−0.17 (−0.30 to −0.05)**	−0.18 (−0.49‐0.12)	−0.15 (−0.47‐0.17)	−0.08 (−1.95‐1.79)

N represents sample size, middle cell values represent *β* (95% CI) of progestin‐only contraceptives, and bottom cell values represent *β* (95% CI) of estrogen‐containing contraceptives compared to no hormonal contraceptive use as the reference group, controlling for covariates.

Abbreviations: CI, confidence interval; CRS, chronic rhinosinusitis.

Bold values denote *P* < .05.

## Discussion

This study examined the relationship between menopause, SHC use, and CRS in adult females. For our study of menopause and CRS, our analysis found that 10.1% of premenopausal women had CRS, compared to 12.1% of postmenopausal women. However, logistic regression analysis revealed no significant difference in CRS odds between postmenopausal and premenopausal individuals after adjusting for covariates. Biomarker analysis revealed that menopause was associated with an elevated concentration of serum eosinophils, particularly among participants without CRS and those with CRSsNP. Literature suggests that estrogen exposure may inhibit eosinophil accumulation and mobilization while also acting as a regulator of prosurvival factors and apoptosis, representing possible mechanisms connecting a decrease of estrogen during menopause with eosinophilia.^27^, ^28^

Biomarker analysis also found that menopause was associated with a lower concentration of serum neutrophils among the full cohort. After stratification by CRS status, this association held for participants with CRSsNP, but was not significant among those with CRSwNP. Literature suggests that estradiol helps delay neutrophil apoptosis, thus a decline in estradiol in menopause may lead to more neutrophil apoptosis, leading to a decline in neutrophil count.^29^ It follows that CRSsNP, which is primarily mediated by a Th1 neutrophilic inflammatory response, should be affected to a greater extent than CRSwNP which utilizes the Th2 eosinophilic inflammatory pathway.^30^ Despite these findings, our analysis did not show a clear independent association between menopause and CRS diagnosis.

For our study of SHC and CRS, we discovered that participants with ECC use had lower odds of having CRSsNP. Some studies provide a possible mechanism for this association, showing that estrogen, particularly estradiol, exerts a protective effect against CRSsNP by suppressing key proinflammatory mediators such as TNF‐α, NK cells, and IFN‐γ. This effect is antagonistic to the chronic inflammation and tissue remodeling found in CRSsNP, which is primarily driven by the neutrophil‐dominated inflammatory pathway and utilizes key cytokines such as IFN‐γ and TGF‐β.^31^ ECC use may not offer this same protective effect for CRSwNP because estrogen promotes the adhesion and recruitment of eosinophils to the nasal mucosa, amplifying the eosinophilic inflammatory pathway that characterizes CRSwNP.^32^ This association could also be related to alterations in sinonasal estrogen receptor expression following contraceptive use, as suggested by some literature.^11^

Statistical analysis also revealed a lack of association between POC use and CRS diagnosis. Current literature is sparse regarding the relationship between progestin and CRS. Researchers have established that progestins act as immunomodulators and suppress proinflammatory cytokine production, downregulating the chronic inflammatory response found in conditions like CRS.^33^ However, it is unclear why CRS odds did not decrease in response to increased POC use in this study. One possible explanation is a lack of progesterone receptors in the sinonasal mucosa, thereby preventing its anti‐inflammatory effects.^8^

Biomarker analysis found that ECC use was associated with a decreased concentration of serum neutrophils among participants without CRS and those with CRSsNP. Despite the protective role of estradiol in delaying neutrophil apoptosis, a different mechanism may play a role when using ECCs in decreasing serum neutrophil count. Studies suggest a few possibilities, including inhibition of production, reduction of neutrophil lifespan, and increased migration of neutrophils out of the plasma and into sites of inflammation.^34^, ^35^ Finally, ECC use was also associated with lower serum uric acid levels among participants without CRS while not being associated among those with CRS. Given the connection between neutrophils and uric acid as biomarkers of non‐type 2 inflammation, these associations align with our main finding that ECC use is associated with a lower odds of CRSsNP.

Our study has several limitations. Its cross‐sectional design prevents us from establishing causation. Socioeconomic factors such as income, education, and insurance status were self‐reported in the AoURP dataset, making them prone to bias. Additionally, CRS diagnoses were based on historical medical records, some of which lacked details on resolution dates. The use of the nasal polyp ICD code for determination of CRSwNP is also a major limitation of this study and may have resulted in misclassification bias, given that CRSwNP patients are often coded without polyp terminology. This may have resulted in an underdiagnosis of participants with CRSwNP, and the inclusion of some participants with CRSwNP in the CRSsNP group. The dataset also did not include information from sinonasal computed tomography or endoscopy, limiting the ability to verify diagnoses. Information regarding surgical interventions for CRS was also absent, limiting our ability to assess treatment history.

Identifying a relationship between menopause, SHC use, and CRS could improve the therapeutic management of this condition through more personalized prevention strategies and treatment approaches for postmenopausal women and individuals using SHCs. Moreover, clinicians could adopt a more proactive approach in recognizing symptoms and implementing focused educational initiatives for high‐risk populations. However, further research is needed to prove the validity of these associations and identify causation. Our findings also underscore the need for continued investigation into the impact of hormone‐modulating physiological states, such as menopause and SHC therapy, on the pathogenesis and progression of CRS.

## Conclusion

In conclusion, our findings revealed no significant association between menopause status or POC use and CRS risk. However, ECC use was independently correlated with a decreased likelihood of CRS, specifically demonstrating a selective association with CRSsNP, while no significant relationship was observed with CRSwNP. Further research should aim to investigate the association of menopause, SHC use and CRS in a longitudinal manner to establish causation and continue to explore mechanisms underlying sex differences in CRS.

## Author Contributions

**Abraham Ahn**, conceptualization, design, conduct, writing—original draft, visualization; **Richard Chiu**, conceptualization, design, conduct, writing—original draft, visualization; **Kamal Eldeirawi**, conceptualization, design, conduct, writing—review and editing, project administration; **Anthony Dick**, conceptualization, design, conduct, writing—review and editing, project administration; **Sharmilee Nyenhuis**, conceptualization, design, conduct, writing—review and editing, project administration; **Victoria Lee**, conceptualization, design, conduct, writing—review and editing, project administration, supervision.

## Disclosures

### Competing interests

None.

### Funding source

Dr Lee is supported by the National Institutes of Health, through Grant Award Number K12AR084225.

## Supporting information


**Table S1**. ICD and SNOMED codes.
**Table S2**. Sample sizes for all CRS biomarkers initially considered for analysis.
**Table S3**. Results of SHC sensitivity analysis removing participants taking contraceptives with low systemic absorption.
**Table S4**. Results of SHC sensitivity analysis removing participants taking drugs listed as estrogen‐only.

**Figure S1**. Sample size flow chart of menopause analysis.

**Figure S2**. Sample size flow chart of SHC analysis.

**Figure S3**. Distribution of time between SHC initiation and CRS diagnosis for participants both taking SHCs and diagnosed with CRS. Each bar represents a 1‐year time range.
