# Supplementary material for: Association of Menopause and Hormonal Contraceptive Use With Chronic Rhinosinusitis: An “All of Us” Analysis
Source: Otolaryngol Head Neck Surg. 2025 Nov 12;174(2):347–58. doi: 10.1002/ohn.70067 (PMC12720269; doi:10.1002/ohn.70067)
Supplement: Supplementary file 1 — Table S1. ICD and SNOMED codes. Table S2. Sample sizes for all CRS biomarkers initially considered for analysis. Table S3. Results of SHC sensitivity analysis removing participants taking contraceptives with low systemic absorption. Table S4. Results of SHC sensitivity analysis removing participants taking drugs listed as estrogen‐only. [file OHN-174-347-s004.docx]

**Table S1**. ICD and SNOMED codes.

| **CRS encompasses the following codes:** | | |
| --- | --- | --- |
| **ICD-9** | **ICD-10** | **SNOMED** |
| 473  Chronic sinusitis | J32  Chronic sinusitis | 40055000  Chronic sinusitis |
| 473.0  Chronic maxillary sinusitis | J32.0  Chronic maxillary sinusitis | 35923002  Chronic maxillary sinusitis |
| 473.1  Chronic frontal sinusitis | J32.1  Chronic frontal sinusitis | 60130002  Chronic frontal sinusitis |
| 473.2  Chronic ethmoidal sinusitis | J32.2  Chronic ethmoidal sinusitis | 73237007  Chronic ethmoidal sinusitis |
| 473.3  Chronic sphenoidal sinusitis | J32.3  Chronic sphenoidal sinusitis | 38961000  Chronic sphenoidal sinusitis |
| 473.8  Other chronic sinusitis | J32.8  Other chronic sinusitis |  |
| 473.9  Chronic sinusitis, unspecified | J32.9  Chronic sinusitis, unspecified |  |
|  |  | 232397007  Chronic frontoethmoidal sinusitis |
|  |  | 427909005  Chronic recurrent sinusitis |
|  |  | 15981951000119106  Chronic right maxillary sinusitis |
|  |  | 15981991000119101  Chronic left maxillary sinusitis |
|  |  | 15981911000119105  Chronic bilateral maxillary sinusitis |
|  |  | 232395004  Chronic panethmoidal sinusitis |
| **CRSwNP was denoted by the presence of one of the following diagnoses:** | | |
| 471  Nasal polyps | J33  Nasal polyp | 736499003  Polyp of nasal cavity and/or nasal sinus |
| 471.0  Polyp of nasal cavity | J33.0  Polyp of nasal cavity | 32307003  Polyp of nasal sinus |
| 471.8  Other polyp of sinus |  | 736500007  Polyp of nasal cavity |
| 471.9  Unspecified nasal polyp | J33.9  Nasal polyp, unspecified | 736636000  Polyp of right nasal cavity |
|  |  | 736635001  Polyp of left nasal cavity |
|  |  | 41931000119102  Sinusitis co-occurrent with nasal polyps |
| **Participants with CRS without a code for CRSwNP were considered to have CRSsNP** | | |

**Table S2**. Sample sizes for all CRS biomarkers initially considered for analysis.

| Biomarker | Menopause Analysis | SHC Analysis |
| --- | --- | --- |
| Serum Eosinophils  (% of WBCs) | 23,519 | 25,549 |
| Serum Neutrophils  (% of WBCs) | 23,222 | 25,277 |
| Serum IgE  (IU/mL) | 943 | 932 |
| Serum Uric Acid  (mg/dL) | 3,868 | 4,344 |
| Serum IFN-γ  (pg/mL) | 0 | 0 |
| Serum TNF-α  (pg/mL) | 4 | 16 |
| Serum IL-4  (pg/mL) | 0 | 0 |
| Serum IL-5  (pg/mL) | 0 | 0 |
| Serum IL-13  (pg/mL) | 0 | 0 |
| Serum IL-17A  (pg/mL) | 0 | 0 |
| Nasal Nitric Oxide  (ppb) | 0 | 0 |
| Urine LTE4  (pg/mg of creatinine) | 4 | 6 |
| Serum MMP9  (ng/mL) | 0 | 2 |

**Table S3**. Results of SHC sensitivity analysis removing participants taking contraceptives with low systemic absorption.

|  | All CRS | CRSsNP | CRSwNP |
| --- | --- | --- | --- |
| Hormonal Contraceptive |  |  |  |
| None | Ref | Ref | Ref |
| Progestin only | 0.88 (0.77–1.01) | 0.87 (0.76–1.00) | 1.09 (0.65–1.73) |
| Estrogen-containing | **0.72 (0.65–0.80)** | **0.71 (0.64–0.80)** | 0.82 (0.51–1.25) |
| Age | **1.05 (1.04–1.05)** | **1.05 (1.04–1.06)** | 1.01 (0.98–1.04) |
| Race/Ethnicity |  |  |  |
| Non-Hispanic White | Ref | Ref | Ref |
| Non-Hispanic Black | **0.68 (0.61–0.77)** | **0.67 (0.59–0.75)** | 1.02 (0.65–1.57) |
| Hispanic | **0.58 (0.53–0.65)** | **0.59 (0.53–0.65)** | **0.57 (0.36–0.87)** |
| Other | **0.56 (0.49–0.64)** | **0.57 (0.50–0.65)** | **0.29 (0.12–0.59)** |
| Annual Income |  |  |  |
| Less than 10,000 | Ref | Ref | Ref |
| 10,000 – 25,000 | 1.14 (0.99–1.31) | 1.12 (0.97–1.29) | 1.52 (0.86–2.71) |
| 25,000 – 35,000 | **1.17 (1.01–1.36)** | 1.15 (0.99–1.34) | 1.52 (0.82–2.82) |
| 35,000 – 50,000 | **1.16 (1.00–1.35)** | 1.16 (0.99–1.35) | 1.19 (0.60–2.33) |
| 50,000 – 75,000 | 1.15 (0.99–1.34) | 1.14 (0.97–1.33) | 1.33 (0.68–2.61) |
| 75,000 – 150,000 | 1.13 (0.97–1.31) | 1.11 (0.95–1.29) | 1.65 (0.87–3.16) |
| More than 150,000 | 0.96 (0.80–1.14) | 0.93 (0.78–1.12) | 1.75 (0.82–3.72) |
| Education |  |  |  |
| Less than high school | Ref | Ref | Ref |
| High school or GED | **1.37 (1.10–1.71)** | **1.38 (1.11–1.73)** | 1.21 (0.49–3.62) |
| Any college | **1.60 (1.30–1.98)** | **1.59 (1.29–1.98)** | 1.75 (0.75–5.11) |
| Graduate/professional | 1.22 (0.97–1.55) | 1.22 (0.97–1.55) | 1.26 (0.48–3.94) |
| Health Insurance, n (%) |  |  |  |
| None | Ref | Ref | Ref |
| Medicare/Medicaid | **1.33 (1.09–1.63)** | **1.31 (1.07–1.61)** | 1.83 (0.75–6.08) |
| Private/Employer | **1.50 (1.23–1.86)** | **1.50 (1.22–1.86)** | 1.60 (0.63–5.40) |
| Other | **1.37 (1.07–1.75)** | **1.36 (1.06–1.75)** | 1.63 (0.51–6.20) |
| Smoking History |  |  |  |
| No | Ref | Ref | Ref |
| Yes | 0.94 (0.86–1.02) | 0.94 (0.86–1.03) | 0.84 (0.57–.21) |
| Asthma |  |  |  |
| No | Ref | Ref | Ref |
| Yes | **2.82 (2.61–3.05)** | **2.71 (2.50–2.93)** | **6.25 (4.54–8.65)** |
| GERD |  |  |  |
| No | Ref | Ref | Ref |
| Yes | **2.90 (2.69–3.13)** | **2.90 (2.68–3.14)** | **2.85 (2.06–3.93)** |

**p < 0.05**

Values represent OR (95% CI)

USD: US Dollars

GED: general educational development

**Table S4**. Results of SHC sensitivity analysis removing participants taking drugs listed as estrogen-only.

|  | All CRS | CRSsNP | CRSwNP |
| --- | --- | --- | --- |
| Hormonal Contraceptive |  |  |  |
| None | Ref | Ref | Ref |
| Progestin only | 0.88 (0.77–1.01) | 0.87 (0.76–1.00) | 1.09 (0.65–1.73) |
| Estrogen-containing | **0.68 (0.61–0.77)** | **0.67 (0.60–0.76)** | 0.84 (0.52–1.30) |
| Age | **1.05 (1.04–1.05)** | **1.05 (1.04–1.06)** | 1.01 (0.98–1.04) |
| Race/Ethnicity |  |  |  |
| Non-Hispanic White | Ref | Ref | Ref |
| Non-Hispanic Black | **0.69 (0.61–0.77)** | **0.67 (0.59–0.76)** | 1.04 (0.66–1.61) |
| Hispanic | **0.59 (0.53–0.65)** | **0.59 (0.53–0.65)** | **0.58 (0.36–0.89)** |
| Other | **0.56 (0.49–0.64)** | **0.57 (0.50–0.65)** | **0.30 (0.13–0.60)** |
| Annual Income |  |  |  |
| Less than 10,000 | Ref | Ref | Ref |
| 10,000 – 25,000 | 1.14 (0.99–1.31) | 1.12 (0.98–1.30) | 1.52 (0.86–2.70) |
| 25,000 – 35,000 | **1.17 (1.01–1.36)** | 1.15 (0.99–1.34) | 1.50 (0.81–2.78) |
| 35,000 – 50,000 | 1.16 (0.99–1.35) | 1.16 (0.99–1.35) | 1.11 (0.55–2.17) |
| 50,000 – 75,000 | 1.15 (0.99–1.34) | 1.15 (0.98–1.34) | 1.26 (0.64–2.48) |
| 75,000 – 150,000 | 1.12 (0.96–1.30) | 1.10 (0.94–1.28) | 1.64 (0.87–3.14) |
| More than 150,000 | 0.95 (0.79–1.14) | 0.93 (0.77–1.11) | 1.76 (0.82–3.73) |
| Education |  |  |  |
| Less than high school | Ref | Ref | Ref |
| High school or GED | **1.36 (1.10–1.70)** | **1.37 (1.10–1.72)** | 1.17 (0.47–3.52) |
| Any college | **1.59 (1.29–1.98)** | **1.59 (1.28–1.72)** | 1.79 (0.77–5.23) |
| Graduate/professional | 1.20 (0.95–1.52) | 1.20 (0.95–1.52) | 1.24 (0.48–3.91) |
| Health Insurance, n (%) |  |  |  |
| None | Ref | Ref | Ref |
| Medicare/Medicaid | **1.33 (1.09–1.63)** | **1.31 (1.07–1.62)** | 1.79 (0.73–5.93) |
| Private/Employer | **1.51 (1.24–1.87)** | **1.51 (1.23–1.87)** | 1.62 (0.64–5.46) |
| Other | **1.38 (1.08–1.78)** | **1.38 (1.07–1.78)** | 1.65 (0.51–6.28) |
| Smoking History |  |  |  |
| No | Ref | Ref | Ref |
| Yes | 0.94 (0.86–1.02) | 0.94 (0.86–1.02) | 0.85 (0.58–1.24) |
| Asthma |  |  |  |
| No | Ref | Ref | Ref |
| Yes | **2.83 (2.62–3.06)** | **2.71 (2.50–2.93)** | **6.48 (4.69–8.98)** |
| GERD |  |  |  |
| No | Ref | Ref | Ref |
| Yes | **2.90 (2.69–3.14)** | **2.90 (2.68–3.14)** | **2.83 (2.04–3.91)** |

**p < 0.05**

Values represent OR (95% CI)

USD: US Dollars

GED: general educational development
