# Supplementary figures and images for: Association of Menopause and Hormonal Contraceptive Use With Chronic Rhinosinusitis: An “All of Us” Analysis
Source: Otolaryngol Head Neck Surg. 2025 Nov 12;174(2):347–58. doi: 10.1002/ohn.70067 (PMC12720269; doi:10.1002/ohn.70067)

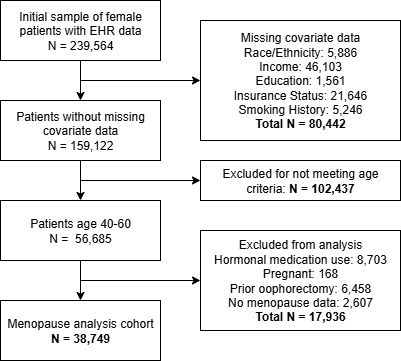

Supplement: Supplementary file 2 — Figure S1. Sample size flow chart of menopause analysis. [file OHN-174-347-s002.tiff]

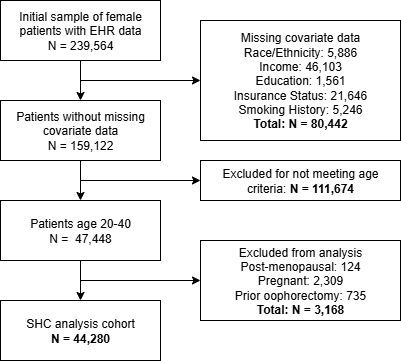

Supplement: Supplementary file 3 — Figure S2. Sample size flow chart of SHC analysis. [file OHN-174-347-s003.tiff]

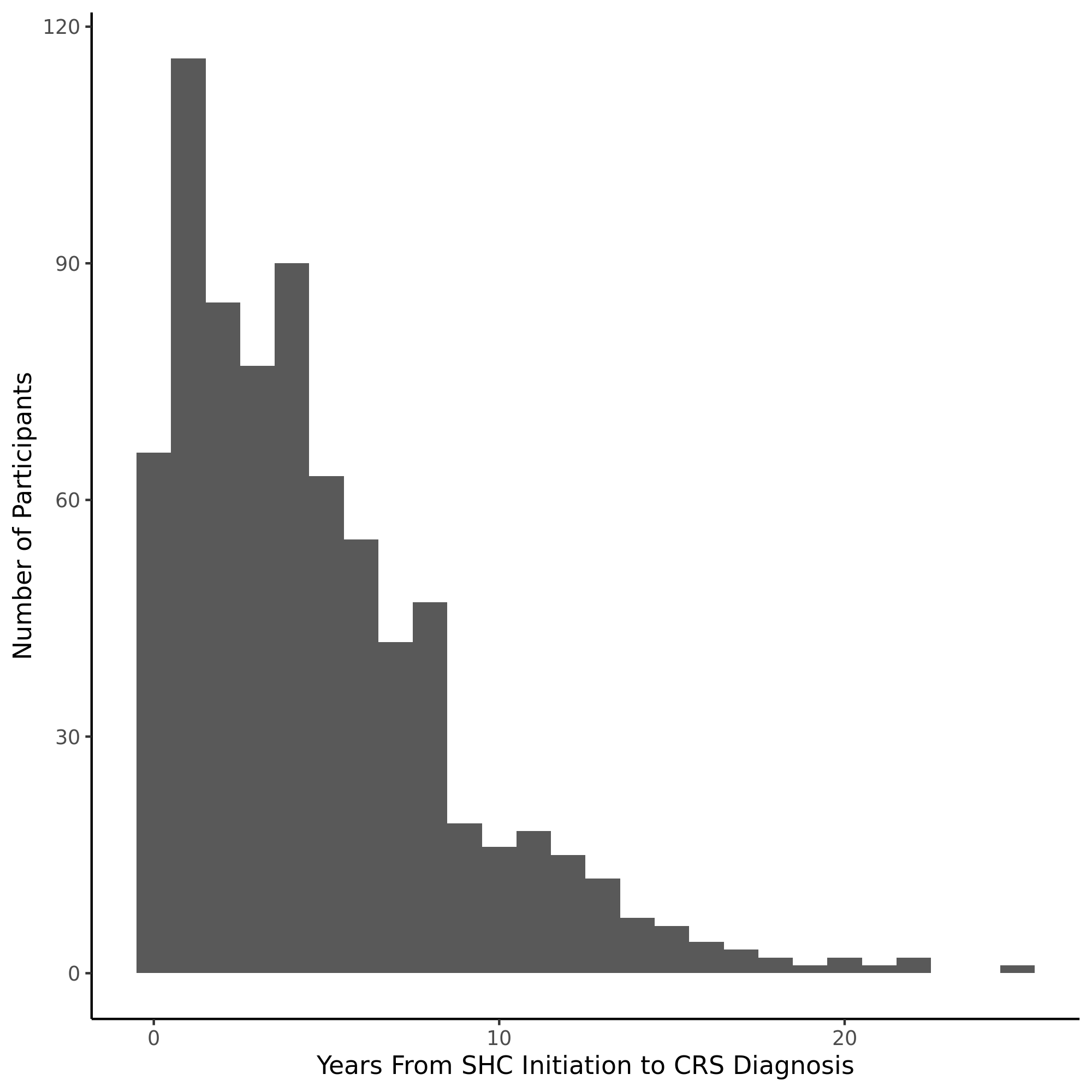

Supplement: Supplementary file 4 — Figure S3. Distribution of time between SHC initiation and CRS diagnosis for participants both taking SHCs and diagnosed with CRS. Each bar represents a 1‐year time range. [file OHN-174-347-s001.tiff]
